# The Inhibitory Effect of Kerra^TM^, KS^TM^, and Minoza^TM^ on Human Papillomavirus Infection and Cervical Cancer

**DOI:** 10.3390/medicina59122169

**Published:** 2023-12-14

**Authors:** Kiattawee Choowongkomon, Khuanjarat Choengpanya, Chamsai Pientong, Tipaya Ekalaksananan, Sulak Talawat, Pussadee Srathong, Jureeporn Chuerduangphui

**Affiliations:** 1 Department of Biochemistry, Faculty of Science, Kasetsart University, Bangkok 10900, Thailand; kiattawee.c@ku.th (K.C.); fscislt@ku.ac.th (S.T.); 2 Program in Basic Science, Maejo University-Phrae Campus, Phrae 54140, Thailand; khuanjarat@mju.ac.th; 3 Department of Microbiology, Faculty of Medicine, Khon Kaen University, Khon Kaen 40002, Thailand; chapie@kku.ac.th (C.P.); tipeka@kku.ac.th (T.E.); 4 HPV & EBV and Carcinogenesis Research Group, Khon Kaen University, Khon Kaen 40002, Thailand; 5 Faculty of Nursing, Praboromarajchanok Institute, Nonthaburi 11000, Thailand; pussadee10@yahoo.com; 6 Department of Microbiology, Faculty of Science, Kasetsart University, Bangkok 10900, Thailand

**Keywords:** herbal medicines, human papillomavirus, HeLa, CaSki, cervical cancer

## Abstract

*Background and Objectives*: Cervical cancer is one of the most common types of frequently found cancers in Thailand. One of the causative agents is the infection of the high-risk human papillomavirus (HPV) type 16 and 18. Traditional medicines are rich sources of bioactive compounds which are a valuable source for the development of novel cancer therapies. In this study, the therapeutic effects of 3 traditional medicines, Kerra^TM^, KS^TM^, and Minoza^TM^, were studied on HeLa and CaSki cells. *Materials and Methods*: The effects of Kerra^TM^, KS^TM^, and Minoza^TM^ on cancer cells were evaluated through cytotoxicity and cell death assays. The infection assay using HPV-16 pseudovirus was also carried out. *Results*: All traditional medicines efficiently suppressed cell growths of HeLa and CaSki, with Kerra^TM^ being the most potent anticancer agent followed by KS^TM^ and Minoza^TM^. Kerra^TM^ at 158 µg/mL and 261 µg/mL significantly increases the percentage inhibition of the HPV-16 pseudovirus infection in a pre-attachment step in a dose-dependent manner, while KS^TM^ at 261 µg/mL efficiently inhibited viral infection in both pre-attachment and adsorption steps. However, Kerra^TM^, KS^TM^, and Minoza^TM^ at subtoxic concentrations could not reduce the viral E6 mRNA expressions of HPV-16 and HPV-18. Cell death assay by acridine orange/ethidium bromide showed that Kerra^TM^ increased population of dead cells in dose-dependent manner in both CaSki and HeLa. The percentage of secondary necrosis in Kerra^TM^-treated CaSki was higher than that of HeLa cells, while the percentage of late apoptotic cells in HeLa was higher than that of CaSki, indicating that HeLa was more susceptible to Kerra^TM^ than CaSki. For KS^TM^ and Minoza^TM^, these extracts at 250 µg/mL promoted autophagy over cell death. At 500 µg/mL, the percentage of dead cells in Kerra^TM^ was higher than that of KS^TM^ and Minoza^TM^. *Conclusions*: Kerra^TM^ is a potent traditional medicine for promoting cancer cell death. Kerra^TM^ is possibly useful in the prevention and treatment of cervical cancer. Further investigation will be carried out to gain a better understanding of the biochemical mechanism and the pharmacological activity underlying this effect.

## 1. Introduction

Cancer is a major public-health burden worldwide. The number of cancer patients are increasing day by day. Cancer cells are the body’s cells that grow uncontrollably and spread to other parts of the body, leading to death. In Thailand, cervical cancer is the second most frequent type of cancer [1]. The cause of cervical carcinoma is infection with high-risk strains of human papillomavirus (HPV). HPV includes many subtypes. Especially, HPV-16 and HPV-18 are the most common subtypes in cervical cancer which can be transmitted sexually [2]. Approximately 80–90% of people infected with HPV will be able to eliminate the infection by their own immunity. However, if the body’s immunity fails to get rid of the HPV infection that occurs over a period of 5–15 years, abnormal changes in the tissue around the cervix can occur and eventually becomes cervical cancer [3].

HPV vaccines are highly effective in the prevention of cervical cancer but have not been widely administered, particularly in low-income and middle-income countries where the number of women who have received a vaccination is <30% [4,5]. Chemotherapeutic drugs, in combination with radiation and surgery used for the treatment of cervical cancer, cause an adverse effect in patients [6]. To minimize the adverse effects of cancer therapy, various natural products have been used to reduce the side effects of these therapies [7]. Currently, plant-derived bioactive compounds have been developed for alternative cancer treatment. They contain diverse active herbal ingredients which have become safe and effective to combine with conventional cancer treatments. For example, in vitro and in vivo studies have shown that the compounds found in *Citrus aurantifolia* (Christm.) Swingle has anticancer activity. Diverse components suspected to have anticancer activity are found in citruses, such as limonoids, flavonoids, essential oils, coumarins, vitamins, and fatty acids [8]. Curcumin and standardized extracts of cultured *Lentinula edodes* mycelia were applied in clinical trials [9,10]. In addition, the prevention of cervical carcinogenesis by a new functional food and food supplement needs to be explored.

As traditional medicine, the effect of Kerra^TM^ on colon cancer has been studied [11]. Kerra™ is an herbal medicine that was developed from the ancient Thai scripture named Tak-Ka-Si-La Scripture that was used as traditional medicine for antipyretics. Kerra™ contains nine medicinal plants, namely *Pterocarpus santalinus* L.f., *Mansonia gagei* J.R.Drumm. ex Prai., *Schumannianthus dichotomus* (Roxb.) Gagnep., *Momordica cochinchinensis* (Lour.) Spreng, *Citrus aurantifolia* (Christm.) Swingle, *Combretum quadrangutare* Kurz, *Tiliacora triandra* (Colebr.) Diels, *Tinospora crispa* (L.) Miers ex Hook.f. and Thoms, and *Dregea volubilis* (l.f.) Hook.f. [12]. Each of these medicinal plants was mentioned in the scripture as having significant potential in cancer therapeutics [13,14,15,16,17]. KS™ is another Thai herbal formulation based on the ancient medical knowledge of the four etiologies of disease which consist of solid, fluid, energy, and gas components. KS™ products are abundant in phytochemical components that consists of a total of twenty plant components, namely *Piper retrofractum* Vahl (fruit), *Zingiber officinale* Roscoe, *Piper sarmentosum* Roxb., *Piper interruptum* Opiz, *Plumbago indica* L., *Mallotus repandus* (Willd.) Mull. Arg., *Anaxagorea luzonensis* A. Gray (wood), *Derris scandens* (Roxb.) Benth, *Piper nigrum* L. (climber), *Ficus foveolata* Wall., *Boesenbergia rotunda* (L.) Mansf., *Senna garrettiana* (Craib), H.S.Irwin and Barneby, *Zingiber cassumunar* Roxb., *Cinnamomum camphora* (L.) J.Presl., *Myristica fragrans* Houtt., *Syzygium aromaticum* (L.) Merr.and L.M. Perry. (flower), *Acorus calamus* L., *Zingiber zerumbet* (L.), *Amomum cardamomum* L. (seed), and *Piper nigrum* L. (seed). Each of these medicinal plants in KS™ exhibits pharmacological properties through various mechanisms. These effects collectively address the prevalent pathogenesis of diseases, alleviate symptoms, and potentially offer medicinal treatment benefits. For example, *Z. officinale* Roscoe or ginger has anti-inflammatories that exhibit pharmacological characteristics similar to those of non-steroidal anti-inflammatory medicines (NSAIDs) [18,19]. Several bioactive components were identified in *M. repandus*, with a particular emphasis on triterpenoids that have been previously documented for their various pharmacological effects, such as anti-inflammatory and anticancer activities [20]. *A. luzonensis* has been widely utilized in Thai traditional medicine for its purported health-promoting properties. It has diverse pharmacological properties, such as antipyretic, stomachic, blood tonic, antioxidant, antihistamine, and antihypertensive properties, along with anti-tyrosinase activity, vasorelaxant impact, and efficacy in treating muscle pain [21]. *D. scandens* is extensively recognized by several vernacular names such “Thao-wan-priang” in Thailand. The findings show that its chemicals have potential for the purposes of cancer prevention and therapy [22]. Thus, these herbal constituents of KS^TM^ exhibit synergistic pharmacological activities, including anti-inflammatory, antibacterial, antioxidative, and anticancer properties. 

Minoza^TM^ is another Thai traditional medicine that contains various herbal constituents. It consists of six plant components, namely *Murdannia loriformis* (Hassk.) R.S. and Kammathy, *Smilax corbularia* Kunth., *Phlogacanthus sirindhorniae* (K.Larsen) Mackinder and R. Clark, *Parinari anamensis* Hance., *Aloe vera* (L.) Burm. F., and *Glycosmis pentaphylla* (Retz.) DC. Each of these components displays distinct characteristics and exhibits pharmacological effects which are utilized for the traditional treatment of detoxification, laxative, antipyretics, sore throat reducer, aphthous ulcer reducer and anticancer. The chemical constituents of these plants comprise of phenols, flavonoids, tannins, alkaloids, and steroids, and they have diverse pharmacological properties such as antioxidants, anti-inflammatories, enhanced immunity, and anticancer effects via several mechanisms including apoptosis, free-radical scavenging, and the prevention of mutation. [23,24,25,26,27,28,29]. For instance, *S. corbularia* Kunth. is rich in flavonoids and saponins, which have effective anti-inflammatory properties [30,31]. The bioactive ingredients of *Glycosmis pentaphylla* (Retz.) DC. have many pharmacological effects including anti-inflammatory, antibacterial, and anticancer properties [32,33,34,35].

The natural mechanism of programmed cell death known as apoptosis is gaining more attention as a target for cancer therapy [36]. The apoptotic pathway is inhibited through several mechanisms, such as overexpression of anti-apoptotic proteins and underexpression of pro-apoptotic proteins that cause mutations and eventually lead to cancer [36]. Plant-derived compounds exhibiting cancer activity through activating the apoptotic pathway have been studied recently [10,11,15,17]. For instance, convincing evidence for the ability of the Kerra™ extract showed that it can activate apoptosis pathway in HCT116 colon-cancer cells, demonstrating their potential as therapeutic agents in this cancer treatment. The extract’s efficacy was demonstrated by its dose-dependent inhibitory effect, induction of apoptotic activity, modulation of key proteins involved in cell death, and proliferation pathways [11]. For KS^TM^ and Minoza^TM^, the anticancer properties have not been studied yet. Therefore, this study aims to investigate the inhibitory effect of Kerra^TM^, KS^TM^, and Minoza^TM^ on HPV infections, growth of cervical cancer cells, and their molecular mechanism.

## 2. Materials and Methods

### 2.1. Cell Cultures

CaSki (HPV-16-positive cervical cancer cell) and HeLa (HPV-18-positive cervical cancer cell) were kindly provided by Prof. Tohru Kiyono (National Cancer Center Research Institute, Japan). A human embryonic kidney 293FT and Vero cells were purchased from Invitrogen (Carlsbad, CA, USA). The cells were cultured in Dulbecco’s Modified Eagle Medium (DMEM; Gibco, NY, USA) containing 10% fetal bovine serum (FBS) and antibiotics (40 µg/mL gentamicin, 2.5 µg/mL amphotericin B, 100 µg/mL streptomycin, and 100 unit/mL penicillin G). The cell culture was incubated in a humidified atmosphere containing 5% CO_2_ at 37 °C.

### 2.2. Herbal Extraction and Cytotoxicity Assay

Kerra^TM^, KS^TM^, and Minoza^TM^ were extracted using 99.5% ethanol. Briefly, 100 g each of Kerra^TM^, KS^TM^, and Minoza^TM^ were mixed with 200 mL of 99.5% ethanol. The mixture was incubated in a shaker at 150 rpm overnight. Then, the extract was filtrated through Whatman no. 1 filter paper and centrifuged at 12,000 rpm at 4 °C for 10 min to remove the precipitate. The ethanol solvent was removed via rotary evaporation at 50 °C. After that, the crude extract was lyophilized. The powder was kept at −20 °C until used.

Vero cells, 293FT, CaSki, or HeLa cell lines at a density of 10,000 cells per well were set up in 96-well plates and incubated for 24 h. The cells were treated with various concentrations of extracts and incubated for 24, 48, 72, and 96 h. Ten microliters of MTT reagent (5 mg/min) were added into the well and continuously incubated for 4 h. A formazan pellet was dissolved in dimethyl sulfoxide (DMSO). The absorbance was measured at 540 nanometers using a spectrophotometer (Multiskan GO, Thermo Fisher Scientific, Vantaa, Finland). The experiments were performed in a triplicate-independent manner.

### 2.3. Anti-HPV-16 Pseudovirus Infection Assay

#### 2.3.1. Production of HPV-16 Pseudovirus

HPV-16 pseudovirus was generated in 293FT co-transfected with p16sheLL (containing HPV-16 L1 and L2 genes) and pfwB (a reporter plasmid) which was kindly provided by John T. Schiller (Laboratory of Cellular Oncology, Bethesda, MD, USA) using Lipofectamine 2000 (Invitrogen, Carlsbad, CA, USA). The co-transfected cells were lysed with a lysis buffer containing 0.5% Brij 58 (Sigma-Aldrich, St. Louis, MO, USA), 0.2% RNase A (bovine pancreas, Sigma Chemical Company, St. Louis, MO, USA), and 9.5 mM MgCl_2_ in PBS after 48 h post-transfection. Concentrated pseudovirus was serially diluted to determine its titer by measuring the total and green-fluorescent cells in 293FT cells under a microscope (Olympus BX51, Olympus, Tokyo, Japan).

#### 2.3.2. Pre-Attachment Step

HPV-16 pseudovirus at the multiplicity of infection (MOI) 0.05 was pre-incubated with each extract of Kerra^TM^, KS^TM^, and Minoza^TM^ for 1 h at 37 °C. The mixture then was added to the 293FT cells and incubated for 4 h at 37 °C. After removing the mixture, the cells were maintained in a complete medium for 48 h. The total and green-fluorescent cells were observed and imaged under a microscope (Olympus BX51, Olympus, Tokyo, Japan). The percentage of inhibition was calculated by subtracting the percentage of infected cells of the treated and control (DMSO) groups.

#### 2.3.3. Adsorption Step

The HPV-16 pseudovirus at the MOI 0.05 was incubated with 293FT cells for 2 h at 20 °C. After removal of the pseudovirus, each extract of Kerra^TM^, KS^TM^, and Minoza^TM^ was transferred to 293FT and incubated at 37 °C for 48 h. The total and green-fluorescent cells were observed imaged under a microscope (Olympus BX51, Olympus, Tokyo, Japan). The percentage of inhibition was calculated by subtracting the percentage of infected cells of the treated and control (DMSO) groups.

#### 2.3.4. RNA

Cervical cancer cells at a density of 60,000 cells per well were loaded into a 24-well plate. Cells were maintained in a complete medium for 24 h, then each extracts at subtoxic concentrations (40 µg/mL for Kerra^TM^ and 120 µg/mL for Kerra^TM^, KS^TM^, and Minoza^TM^) was mixed with the cells for 48 h. After harvesting, the cells were subjected to Trizol reagent (Invitrogen, Carlsbad, CA, USA), and subsequently chloroform. The upper and lower phase were separated by centrifugation and aliquoted into a new tube. The upper one was mixed with isopropanol to precipitate RNA. The RNA pellet was dissolved in nuclease-free water and kept at −80 °C.

#### 2.3.5. HPV-16/18 E6 mRNA Expression

Total RNA was used as a template for cDNA synthesis using Revert Aid First Strand cDNA Synthesis Kit (Thermo Fisher Scientific, Waltham, MA, USA) with an oligo dT primer. Diluted cDNA was subjected to SYBR Green (Bio-Rad, Hercules, CA, USA) and a specific primer to determine the HPV-16 E6 and HPV-18 E6 gene expression. The amplification was conducted in an Eco48 real-time qPCR system (PCRmax, Staffordshire, UK).

### 2.4. Cell Death Assay by Acridine Orange/Ethidium Bromide

CaSki and HeLa at a density of 60,000 cells were seeded into each well of a 24-well plate and maintained in a complete medium for 24 h. Kerra^TM^, KS^TM^, and Minoza^TM^ at concentrations of 250 and 500 μg/mL were mixed with the cells in each well and continuously incubated for 48 h. After harvesting, the cells were stained with each 100 μg/mL of acridine orange and ethidium bromide (AO/EB) (Sigma-Aldrich, St. Louis, MO, USA). The cell morphology was observed under fluorescent microscopy (Olympus BX51, Olympus Co., Ltd., Tokyo, Japan), and distinguished according to Supplementary Figure S1.

## 3. Results

### 3.1. The Effect of Kerra^TM^, KS^TM^, and Minoza^TM^ Extracts on Cytotoxicity In Vero Cells

The cytotoxic activity study of Kerra^TM^, KS^TM^, and Minoza^TM^ on Vero cells at several concentrations ranging from 3.91–500 µg/mL revealed that Kerra^TM^ and Minoza^TM^ showed low cytotoxicity against Vero cells with CC50 values higher than 500 µg/mL, respectively (Supplementary Figure S2A,B). KS^TM^ was slightly cytotoxic to Vero cells with an CC50 value of 363.3 µg/mL (Supplementary Figure S2C). From these results, it could be said that Kerra^TM^, KS^TM^, and Minoza^TM^ had low cytotoxic effects on Vero cells.

### 3.2. The Effect of Kerra^TM^, KS^TM^, and Minoza^TM^ Extracts on Cytotoxicity in 293FT, CaSki and HeLa

Kerra^TM^ showed the highest efficacy in inhibiting the growth of CaSki and HeLa cells, followed by KS^TM^ and Minoza^TM^ in a dose- and time-dependent manner (Table 1). Similar to cancerous cell lines, Kerra^TM^ also showed the highest cytotoxicity in 293FT (Table 1).

### 3.3. The Effect of Kerra^TM^, KS^TM^, and Minoza^TM^ Extracts on Anti-HPV-16 Pseudovirus Infection

**Figure 1:**
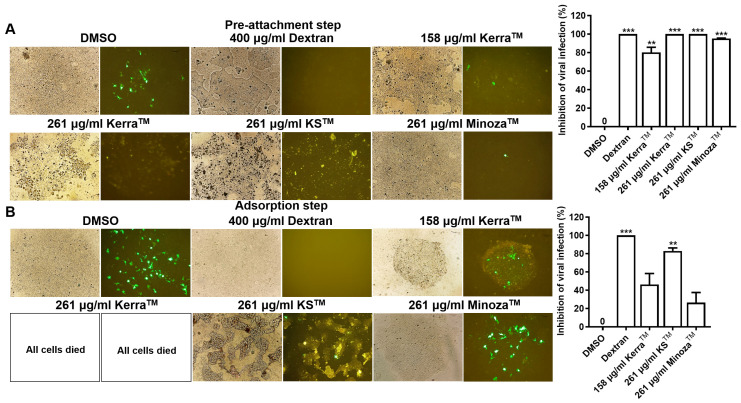
Effect of Kerra^TM^, KS^TM^, and Minoza^TM^ on anti-HPV-16 pseudovirus infection assay. The extract of Kerra^TM^, KS^TM^, and Minoza^TM^ were assessed on anti-HPV-16-pseudovirus infections in the pre-attachment step (**A**) and adsorption step (**B**). The symbols ** and *** were denoted as the significant difference (*p* ≤ 0.01 and 0.001, respectively).

From Table 1, the Kerra^TM^ extract most effectively suppressed the cell viability of CaSki and HeLa. Therefore, the CC20 (158 μg/mL) and CC50 (261 μg/mL) of the Kerra^TM^ extract were used to assess the anti-HPV-16 pseudovirus infection in 293FT cells and to compare with KS^TM^ and Minoza^TM^. The result showed that Kerra^TM^ significantly increased the percentage inhibition of HPV-16 pseudovirus infection in the pre-attachment step in a dose-dependent manner, with CC20 and CC50 values of 80.15 ± 5.56 and 100 ± 0.00, respectively (Figure 1A). Simultaneously, Kerra^TM^ showed the trend of elevating percentage inhibition in the adsorption step (Figure 1B). To compare the effect of Kerra^TM^, KS^TM^, and Minoza^TM^ on anti-HPV-16 pseudovirus infections, the concentration of KS^TM^ and Minoza^TM^ was 261 μg/mL, similar to Kerra^TM^ at CC50. Interestingly, KS^TM^ showed the highest inhibition of viral infection in the pre-attachment and adsorption steps with percentage inhibition values of 100 ± 0.00 and 82.94 ± 3.28, respectively.

### 3.4. Kerra^TM^, KS^TM^, and Minoza^TM^ Could Not Suppress Viral Oncogene at Subtoxic Concentrations

**Figure 2:**
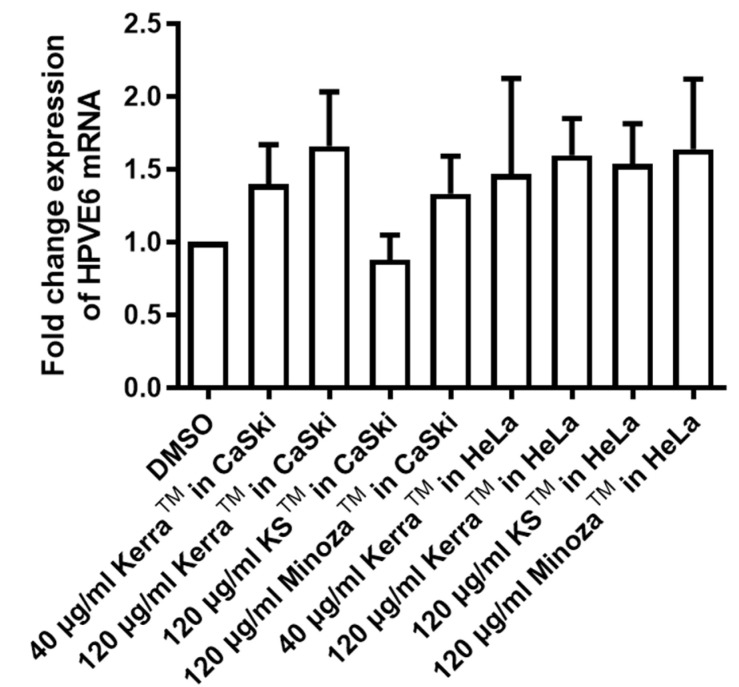
HPV-16 E6 and HPV-18 E6 mRNA expression in Kerra^TM^, KS^TM^, and Minoza^TM^ treatment in CaSki and HeLa. Either Kerra^TM^, KS^TM^, or Minoza^TM^ at 40 and 120 μg/mL was mixed with CaSki and HeLa cells for 48 h. The expression levels of HPV-16 E6 and HPV-18 E6 mRNAs were analyzed using cycle thresholds to calculate the fold change expression compared with DMSO (control).

Kerra^TM^, KS^TM^, and Minoza^TM^ at subtoxic concentrations (40 µg/mL and 120 µg/mL: ≤CC50) were used to study their effects on anti-viral oncogene expression. CaSki and HeLa treated with each of the three extracts could not reduce the viral oncogene E6 mRNA expression (Figure 2). Notably, the effects of the increased concentration of these extracts on the viral oncogene E6 mRNA expression need to be further investigated.

### 3.5. Kerra^TM^-, KS^TM^-, Minoza^TM^-Promoted Cell Death in CaSki and HeLa

**Figure 3:**
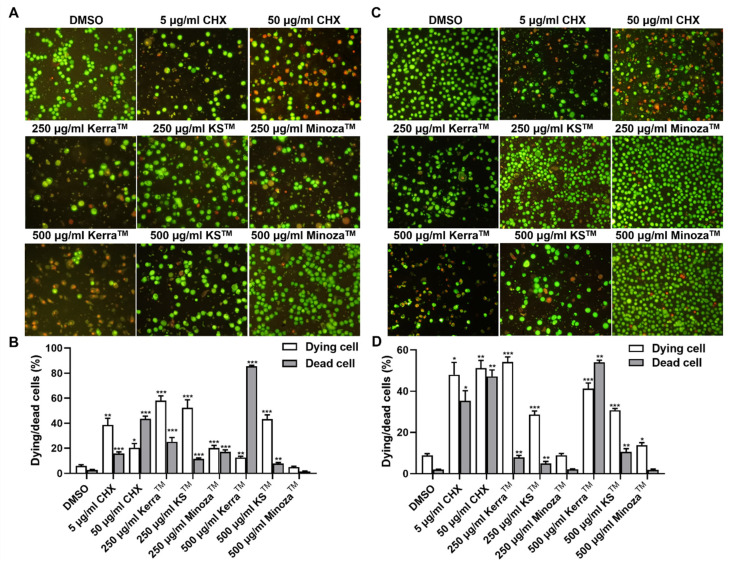
Cell morphology of Kerra^TM^, KS^TM^, and Minoza^TM^-treated CaSki and HeLa stained with AO/EB. The morphology of each Kerra^TM^, KS^TM^, and Minoza^TM^-treated CaSki (**A**) and HeLa (**C**) dual stained with AO/EB was imaged under a fluorescent microscope. Percentages of dying and dead cells in CaSki (**B**) and HeLa (**D**) were measured from the images taken under the microscope. The symbols *, ** and *** were denoted as the significant difference (*p* ≤ 0.05, 0.01 and 0.001, respectively).

Because Kerra^TM^, KS^TM^, and Minoza^TM^ at subtoxic concentration (≤CC50) could not reduce mRNA level of HPVE6 oncogene, the concentrations of these extracts were increased to evaluate a population of cell death by AO/EB. In order to compare the effect of Kerra^TM^ on cell death, KS^TM^ and Minoza^TM^ were used at the same concentration. Kerra^TM^ at 250 μg/mL had nearly an equal CC50 in 48 h and mostly increased the dying or dead CaSki cells, followed by KS^TM^ and Minoza^TM^ as shown in Table 1 and Figure 3. Only Kerra^TM^ showed an increase in dead cells in a dose-dependent manner. Interestingly, Kerra^TM^ (53.59 ± 2.87%), KS^TM^ (48.09 ± 6.62%), and Minoza^TM^ (15.41 ± 1.64%) at 250 μg/mL promoted higher autophagy than dead cells in contrast to the control (3.11 ± 0.38%). When the concentration increased to 500 μg/mL, the autophagic cells in Kerra^TM^ decreased while the dead cells were elevated, and was higher than the first one. Simultaneously, the 250 and 500 μg/mL concentrations of Kerra^TM^ were more toxic to HeLa for 48 h at the CC50, significantly increasing the population of dying and dead cells compared to KS^TM^ and Minoza^TM^ (Table 1, Figure 3C,D). Similar to CaSki, Kerra^TM^ increased a population of dead cells in a dose-dependent manner in HeLa. However, Kerra^TM^ induced a percentage of late apoptotic cells in HeLa with 250 and 500 μg/mL of 5.10 ± 0.42% and 23.08 ± 1.51%, respectively, which were higher than those of CaSki (250 and 500 μg/mL of 2.03 ± 0.85% and 0.72 ± 0.25%, respectively). The population of secondary necrosis in treated CaSki (250 and 500 μg/mL of 18.69 ± 3.10% and 74.43 ± 6.16%, respectively) was higher than that of HeLa (250 and 500 μg/mL of 2.19 ± 0.59% and 30.22 ± 0.94%, respectively). These results demonstrated that HeLa was more susceptible to the Kerra^TM^ treatment than CaSki in promoting late apoptosis. These results indicate that Kerra^TM^ is a potential drug for promoting cancer cell death.

## 4. Discussion

High-risk HPV subtypes, especially HPV-16 and HPV-18, are the most common cause of cervical cancer [2]. These two types of HPV cause 70% of invasive cervical cancers in the world [2]. Thus, CaSki (HPV-16-positive cervical cancer cells) and HeLa (HPV-18-positive cervical cancer cells) were selected to study the effect on antiviral oncogene expression.

The treatment of cervical cancer through chemotherapy and radiotherapy causes adverse side effects [6]. The HPV vaccination is highly effective in preventing cervical cancer, however, access to HPV vaccinations in low- and middle-income countries is still limited. The application of natural products, which are rich sources of anticancer substances, in cervical cancer treatment is a promising and alternative approaches for cervical cancer treatment [8,9]. 

Cytotoxicity studies of Kerra^TM^, KS^TM^, and Minoza^TM^ revealed that these extracts showed a low toxicity against Vero cells. Interestingly, Kerra^TM^ showed the highest suppression of cell viability in HPV-positive cervical cancer cells, followed by KS^TM^ and Minoza^TM^ in a dose- and time-dependent manner. Similar to cancerous cell lines, Kerra^TM^ caused the highest cytotoxicity in 293FT (Table 1). Additionally, Kerra^TM^ completely prevented HPV-16 infection in the pre-attachment step whereas KS^TM^ effectively prevented viral infection in both the pre-attachment and adsorption steps (Figure 1A,B). Moreover, the study of the effect of Kerra^TM^, KS^TM^, and Minoza^TM^ on cell death demonstrated that HeLa was more susceptible to Kerra^TM^ than CaSki. Kerra^TM^ promoted late apoptosis in HeLa cells (Figure 3). Though the efficiency of Kerra^TM^ on the inhibition of HPV-16 infection was lower than that of KS^TM^ (Figure 2), its effect was mostly on the growth suppression of cervical cancers in comparison with KS^TM^ and Minoza^TM^. It can be proposed that Kerra^TM^ is a possible candidate for cervical cancer prevention and treatment. Notably, increased concentrations of these extracts need to be further investigated for cell viability suppression on HPV-positive cervical cancer cells.

Kerra^TM^ comprises of nine medicinal plants, in which some of their phytochemicals have been studied to have anti-inflammatory and anticancer properties [11]. These phytochemicals affect various signaling pathways, including anti-inflammation, cell proliferation, and apoptosis [8,10,37,38,39,40,41,42]. For example, the heartwood of *P. santalinus* L.f. contains various bioactive compounds, e.g., pterostilbene, which has been shown to be potent against cervical cancer. It inhibited the growths of HeLa and CaSki cells with IC_50_ values of 32.67 and 14.83 µM, respectively. Pterostilbene induced cell-cycle arrest by increasing the expression levels of p53 and p21 and decreasing the expression levels of cyclin E1 and cyclin B1. In addition, it induced apoptosis through the activation of caspase-3 and caspase-9, production of reactive oxygen species (ROS), downregulation of the Bcl-2 and Bcl-XL anti-apoptotic proteins, as well as the inhibition of MMP-2 and MMP-9 expressions [38]. Coumarins and *O*-Naphthoquinones from the heartwood of *Mansonia gagei* J.R.Drumm. ex Prai., such as mansorin-A, mansorin-B, mansorin-C, mansorin II, mansorin-I, and mansonone-G have anti-cervical cancer activity with IC_50_ values against HeLa cells ranging between 0.74–18.8 µM [39]. Coumarin has been found to induce cell-cycle arrest and apoptosis in HeLa cells via a decrease in the expression of G0/G1-associated proteins and the Bcl-xL and Bcl-2 anti-apoptotic proteins, but it increases the expression of the pro-apoptotic protein Bax [37]. Coumarin also decreases the mitochondrial membrane potential, leading to the release of cytochrome c, and the activation of caspase-3 and apoptosis [39]. Momardin Ic, a saponin compound commonly found in the root of *M. cochinchinensis* (Lour.) Spreng [40], showed anti-cell proliferative activity in cervical cancer [41]. Several phytochemicals in *Citrus aurantifolia* (Christm.) Swingle such as limonoids, phenolic acids (gallic acid and ferulic acid), and flavones (hesperetin and naringenin) have been shown to be anti-cervical cancer agents through various pathways. [10,41,42]. Ferulic acid has been shown to inhibit HeLa and CaSki cell proliferation and invasion via under-expression of MMP-9 mRNA, induction of G0/G1 cell arrest, as well as the inhibition of autophagy [42]. For limonoids, though there is no evidence of anti-cervical cancer properties, it has been shown to inhibit colon, stomach, and breast cancers by inhibiting cell proliferation and caspase-mediated apoptosis [10,42]. Hesperetin induces apoptosis through upregulations of caspases, p53, Bax, and Fas death receptors, while naringenin inhibits the growth of HeLa cells and induces apoptosis through the inhibition of the NF-κB/COX-2/caspase-1 pathway. Naringenin also induces G1 cell-cycle arrest via induction of the p21WAF1 expression, which subsequently leads to a decrease in the levels of the cyclin D1/CDK4 and cyclin E-CDK2 complexes and cell growth [42]. *C. quadrangulare* Kurz is a rich source of alkaloids and triterpenes, but their anti-cervical cancer activity has not been studied yet. Combretin, a steroidal alkaloid isolated from the seeds of *C. quadrangulare* Kurz, showed anticancer activities against human hepatocarcinoma (Hep G2) ATCC HB-8065 and human Caucasian colon adenocarcinoma (Caco2) ATCC HTB-39 [43]. Combretic acid C, a triterpene isolated from the leaves of *C. quadrangulare* Kurz has strong cytotoxicity against the K562 cancer cell line with an IC_50_ value of 9.7 µM [44]. Combretastins A-1 and A-4 prodrugs from the related species of *C. quadrangulare* Kurz, *C. caffrum*, are currently investigated in Phase I human cancer clinical trials [45]. *T. triandra* (Colebr.) Diels extracts comprise of many bioactive compounds with anticancer properties, including p-coumaric, ferulic acid, sinapic acid, and phytol [46,47]. The methanolic extract from the leaves of *T. triandra* (Colebr.) Diels has been tested with HeLa cells and the IC_50_ value of the cell viability was 0.41 mg/mL [47]. The chemical constituents in *Tinospora crispa* (L.) Hook. f. and Thomson have been studied extensively, and various chemicals such as crispenes C, D, F, and G showed cytotoxicity against STAT3-dependent MDA-MB 231 breast cancer cells [48]. Though anti-cervical cancer substances have not been investigated yet, the aqueous, methanol, and chloroform extracts of stems showed cytotoxicity against HeLa cells with IC50 values of 53.83 ± 1.47, 52.5 ± 1.14, and 46.13 ± 2.81 µg/mL, respectively [49]. The final medicinal plant component in Kerra^TM^ is *Dregea volubilis* (L.f.) Hook.f. It consists of several bioactive compounds, e.g., apigenin, isoorientin, luteolin, quercetin, rutin, β-sitosterol, kaempferol, et cetera [50]. The methanolic extract of its leaves showed a cytotoxic effect against HeLa cells with a 50% net killing value (CTC_50_) of 210 µg/mL [51].

Though the specific biochemical mechanisms or pharmacological activities underlying these effects in the current study are not yet thoroughly understood, the results of this current study suggested that Kerra^TM^ is potentially useful in cervical cancer prevention and treatment. To gain a better understanding of the effect of Kerra^TM^ on growth suppression of cervical cancers, its overall mechanisms such as anti-angiogenesis, anti-metastasis, or drug resistance will be further investigated. Moreover, to apply Kerra^TM^ in the treatment of cervical cancer, sufficient clinical studies are required to confirm its clinical safety and efficiency. Further investigations will be focused on its purification and pharmacokinetics, and identification of components for cervical cancer treatment are essential to achieve this goal.

In addition, the effect of a combination formula of Kerra^TM^, KS^TM^, and Minoza^TM^ is also needed to determine the appropriate proportion of these three extracts on HPV infection and cervical cancer. Preclinical and clinical studies of these formulas are also needed.

This current study provides a first foundation for the possible therapeutic impact of Thai herbal medicine on the growth suppression of cervical cancers. It is well known that complementary medicine is also extensively used among cancer patients worldwide [52]. In addition, several studies have indicated that herbal medicine is the most common form of complementary and alternative medicine (CAM) used by patients with cancer, with increasing use following a cancer diagnosis [53,54,55,56,57,58,59,60,61]. Some conventional treatments of cancer tend to have severe side effects, drug resistance, multiple recurrences, and metastases that cause considerable suffering to patients. Therefore, this current study reports novel traditional medicines with a high efficacy and low cytotoxicity towards normal cells. They can be utilized as a complementary medicine or adjuvant treatment to either help relieve some side effects of conventional cancer treatment or prevent and treat cancer, especially cervical cancer.

## 5. Conclusions

The anti-cervical activities of three Thai traditional medicines, namely Kerra^TM^, KS^TM^, and Minoza^TM^ (the trademarks of the products), were investigated. Kerra^TM^, which was developed from the Tak-Ka-Si-La Scripture, efficiently suppressed the cell growth of HeLa and CaSki cells. Kerra^TM^ also significantly increased the percentage inhibition of HPV-16 pseudovirus infections in the pre-attachment step. KS^TM^ and Minoza^TM^ could also suppress cancer cell growth. KS^TM^ efficiently inhibited viral infections in both the pre-attachment and adsorption steps. However, all extracts at subtoxic concentrations (40 µg/mL and 120 µg/mL: ≤CC50) could not reduce the viral E6 mRNA expressions of HPV-16 and HPV-18. Cell death analysis revealed that Kerra^TM^ promoted secondary necrosis in CaSki cells and late apoptosis in HeLa cells. It increased the population of dead cells in a dose-dependent manner in both cancer cells. KS^TM^ and Minoza^TM^ promoted autophagy over cell death. The effect of Kerra^TM^ was mostly growth suppression in comparison with KS^TM^ and Minoza^TM^. Kerra^TM^ is a possible candidate for cervical cancer prevention and treatment. Further investigations on their pharmacokinetics as well as preclinical and clinical studies will be carried out to better understand their mechanisms and safety use in cervical cancer treatment.

## Figures and Tables

**Table 1:** The cytotoxic concentration of 293FT, Kerra^TM^, KS^TM^, and Minoza^TM^.

Extract	Incubation Time (Hours)	Cytotoxic Concentration (mg/mL) of 293FT	
		CC20	CC50
Kerra^TM^	48	0.15 ± 0.01	0.26 ± 0.04
KS^TM^	48	0.41 ± 0.18	1.91 ± 0.68
Minoza^TM^	48	1.97 ± 0.16	5.20 ± 0.04
**Extract**	**Incubation Time** **(Hours)**	**CC50 (mg/mL)**	
		**CaSki**	**HeLa**
Kerra^TM^	24	0.33 ± 0.12	0.16 ± 0.08
	48	0.21 ± 0.02	0.12 ± 0.07
	72	0.11 ± 0.03	0.08 ± 0.03
	96	0.09 ± 0.01	0.07 ± 0.02
KS^TM^	24	>2	>2
	48	1.82 ± 0.05	0.62 ± 0.18
	72	0.72 ± 0.14	0.58 ± 0.18
	96	0.71 ± 0.32	0.31 ± 0.19
Minoza^TM^	24	>2	>2
	48	1.19 ± 0.15	1.17 ± 0.14
	72	0.96 ± 0.14	0.60 ± 0.07
	96	0.93 ± 0.08	0.33 ± 0.11

Note: The data are expressed in mean ± standard deviation.

## Data Availability

Upon reasonable request, the corresponding author is willing to provide the data and materials supporting the results of this study.

## Supplementary Materials

The following supporting information can be downloaded at: https://www.mdpi.com/article/10.3390/medicina59122169/s1, Figure S1: The criteria to distinguish cell morphology in acridine orange/ethidium bromide staining of CaSki and HeLa cells; Figure S2: Cytotoxic effects of Kerra^TM^, Minoza^TM^, and KS^TM^.
