# Supplementary material for: The Inhibitory Effect of KerraTM, KSTM, and MinozaTM on Human Papillomavirus Infection and Cervical Cancer
Source: Medicina (Kaunas). 2023 Dec 14;59(12):2169. doi: 10.3390/medicina59122169 (PMC10745032; doi:10.3390/medicina59122169)
Supplement: Supplementary file 1 [file medicina-59-02169-s001.zip › medicina-2747559-supplementary.pdf]

The inhibitory effect of Kerra™, KS™, Minoza™ on human papillomavirus infection and cervical cancer

Kiattawee Choowongkomon [1,†], Khuanjarat Choengpanya [2,†], Chamsai Pientong [3,4], Tipaya Ekalaksananan [3,4],
Sulak Talawat [1], Pussadee Srathong [5] and Jureeporn Chuerduangphui [6,*]

[1] Department of Biochemistry, Faculty of Science, Kasetsart University, Bangkok 10900, Thailand; kiattawee.c@ku.th (K.C.); fscislt@ku.ac.th (S.T.)
[2] Program in Basic Science, Maejo University-Phrae Campus, Phrae 54140, Thailand; khuanjarat@mju.ac.th
[3] Department of Microbiology, Faculty of Medicine, Khon Kaen University, Khon Kaen 40002, Thailand; chapie@kku.ac.th (C.P.); tipeka@kku.ac.th (T.E.)
[4] HPV & EBV and Carcinogenesis Research Group, Khon Kaen University, Khon Kaen 40002, Thailand
[5] Faculty of Nursing, Praboromarajchanok Institute, Nonthaburi 11000, Thailand; pussadee10@yahoo.com
[6] Department of Microbiology, Faculty of Science, Kasetsart University, Bangkok 10900, Thailand
* Correspondence: fscijoc@ku.ac.th; Tel.: +66-862182348
† These authors contributed equally to this work.

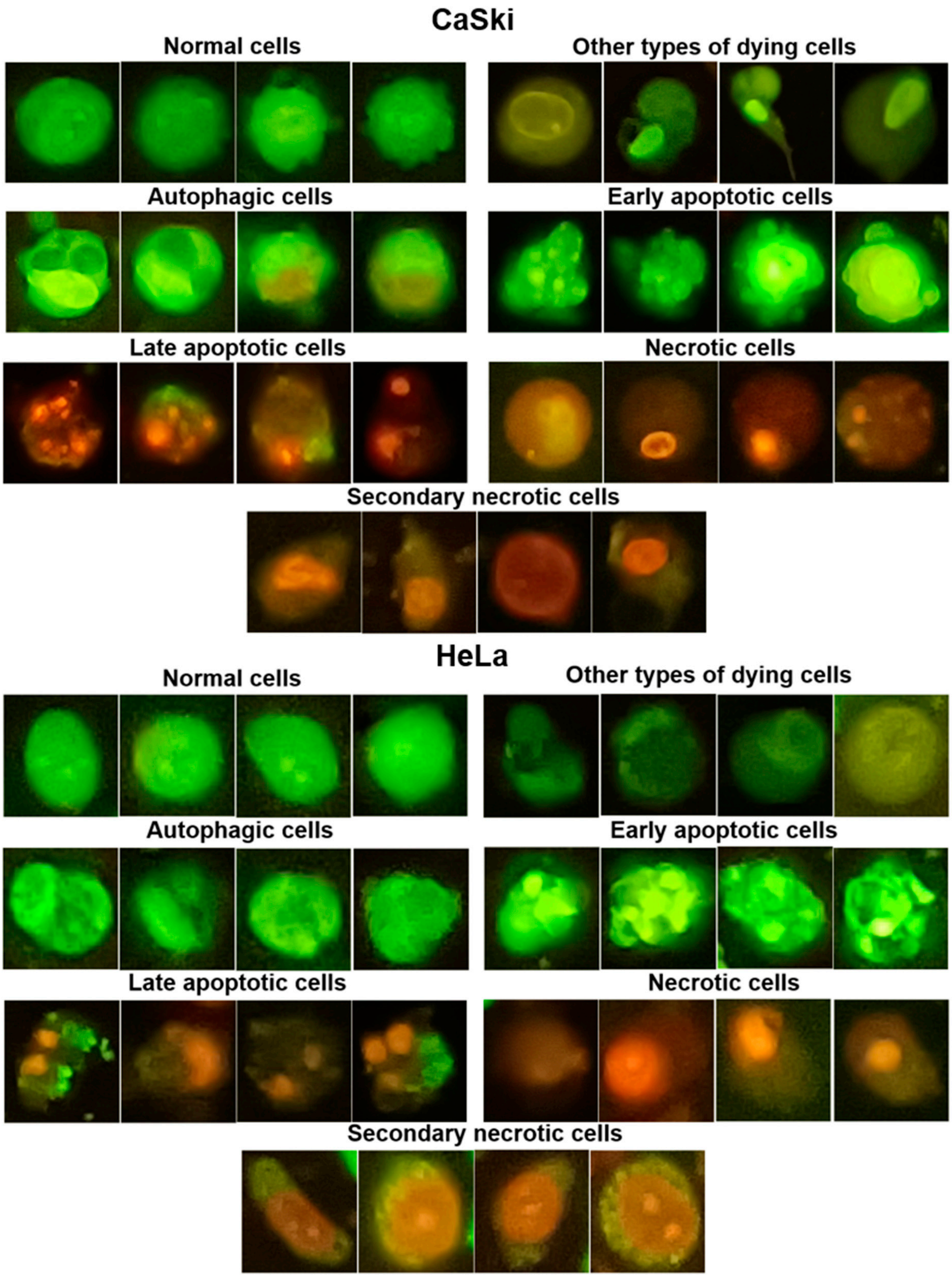

Supplementary Figure S1. The criteria to distinguish cell morphology in acridine orange/ethidium bromide staining of CaSki and HeLa cells. Green-fluorescent cells with normal nucleus and cytoplasm characteristics represent normal cells. Green or yellow cells with abnormal nucleus and cytoplasm differenced from early apoptotic and phagocytic cells represent dying cells. Early apoptotic cells are characterized by green or yellow cells with chromosome condensed and cell membrane bubbling. Orange or red expressing cells with chromatin condensed are late apoptotic cells. Swelling of cytoplasm and abnormal size of cytoplasm are necrotic cells. Finally, orange, or red nucleus with shrunken or normal cytoplasm is secondary necrosis.

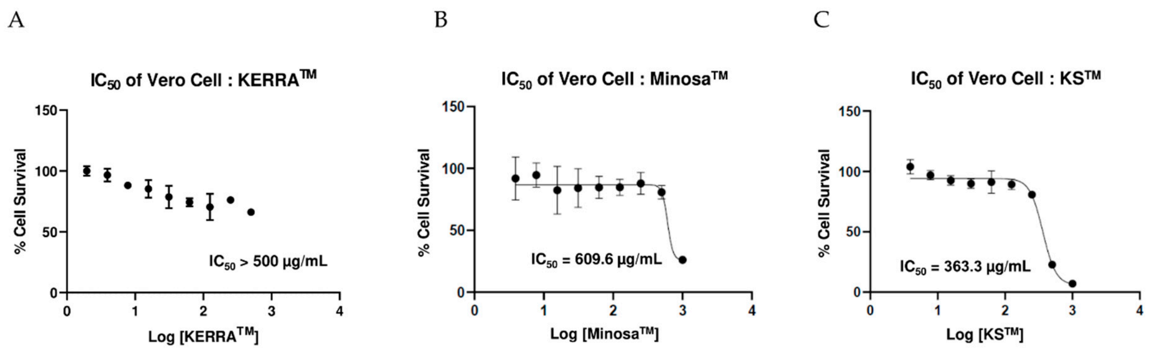

Supplementary Figure S2. Cytotoxic effects of Kerra™ (A), Minosa™ (B), and KS™ (C) against Vero cells after 72 h of exposure using MTT assay at the concentrations ranging from 3.91 – 500 µg/ml in logarithmic scale.
